# Nogo-C regulates post myocardial infarction fibrosis through the interaction with ER Ca^2+^ leakage channel Sec61α in mouse hearts

**DOI:** 10.1038/s41419-018-0598-6

**Published:** 2018-05-23

**Authors:** Lin Weng, Shi Jia, Chunling Xu, Jingjing Ye, Yangpo Cao, Yingying Liu, Ming Zheng

**Affiliations:** Key Laboratory of Molecular Cardiovascular Science, Ministry of Education, Beijing, China

## Abstract

Cardiac fibrosis is an independent risk factor for heart failure and even the leading cause of death in myocardial infarction patients. However, molecular mechanisms associated with the pathogenesis of cardiac fibrosis following myocardial infarction are not yet fully understood. Nogo-C protein ubiquitously expresses in tissues including in the heart. Our previous study found that Nogo-C regulated cardiomyocyte apoptosis during myocardial infarction. In the present study, we found that Nogo-C was upregulated in fibrotic hearts after myocardial infarction and in Ang II- or TGF-β1-stimulated cardiac fibroblasts. Overexpression of Nogo-C in cardiac fibroblasts increased expression of pro-fibrogenic proteins, while knockdown of Nogo-C inhibited the fibrotic responses of cardiac fibroblasts to Ang II- or TGF-β1 stimulation. Functionally, Nogo-C deficiency suppressed pro-fibrogenic proteins in post-myocardial infarction hearts and ameliorated post-myocardial infarction cardiac function. Mechanistically, we found that Nogo-C increased intracellular Ca^2+^ concentration and buffering Ca^2+^ totally abolished Nogo-C-induced fibrotic responses. Moreover, overexpression of Nogo-C caused increased Sec61α, the Ca^2+^ leakage channel on endoplasmic reticulum membrane. Nogo-C interacted with Sec61α on endoplasmic reticulum and stabilized Sec61α protein by inhibiting its ubiquitination. Inhibition or knockdown of Sec61α blocked Nogo-C-induced increase of cytosolic Ca^2+^ concentration and inhibited Nogo-C- and TGF-β1-induced fibrotic responses in cardiac fibroblasts, suggesting that Nogo-C regulates cardiac fibrosis through interacting with Sec61α to mediate the Ca^2+^ leakage from endoplasmic reticulum. Thus, our results reveal a novel mechanism underlying cardiac fibrosis following myocardial infarction, and provide a therapeutic strategy for cardiac remodeling related heart diseases.

## Introduction

Following myocardial infarction (MI), inflammatory cells such as neutrophils and macrophages recruit to the injured area to clear cardiomyocyte debris and secrete inflammatory cytokines^1^. The elevated inflammatory cytokines and growth factors including transforming growth factor-β (TGF-β) then recruit and activate myofibroblasts. Mainly differentiated from fibroblasts, activated myofibroblasts secrete a large amount of extracellular matrix (ECM), such as fibronectin (FN) and collagen, to replace the lost heart tissue, leading to structural and molecular remodeling of the heart^2,3^. Although the replacement fibrotic remodeling at early stage protects the heart from ventricular rupture, adverse remodeling leads to ventricular wall stiffness, impaired cardiac compliance, and consequently decreased cardiac contraction and/or diastolic function^2,4–6^. Indeed, adverse cardiac fibrosis is an important independent risk factor for heart failure and is the leading cause of death in MI patients^7^. Thus, understanding molecular mechanisms underlying cardiac fibrosis is of great importance to the treatment of patients at high risk of developing heart failure after MI.

Multiple pro-fibrotic molecules are involved in the pathogenesis of cardiac fibrosis. TGF-β/Smad signals are essential players in the differentiation of myofibroblast and interstitial deposition of ECM proteins after MI. For instance, TGF-β-mediated production of ECM in cardiac fibroblasts is Smad3-dependent^5,8^. Smad3 also regulates the promoter activity of connective tissue growth factor (CTGF), a key mediator of ECM production during cardiac fibrotic remodeling^9^. Smad-independent signals such as p38 mitogen-activated protein kinase (MAPK) have also been associated with the profibrotic TGF-β response^10^. In addition to TGF-β, renin-angiotensin system plays an important role in the cardiac remodeling. Activated myofibroblasts express angiotensin II (Ang II), the central part of the renin-angiotensin system, and elevated Ang II consequently regulates the differentiation of myofibroblasts or the expression of ECM proteins. In fact, TGF-β pathway is directly associated with renin-angiotensin system by serving as the downstream of Ang II. Emerging evidence have shown that Ca^2+^ signals contribute to Ang II-induced fibrosis process. Chronic administration of Ang II in rat increases cytoplasm Ca^2+^, induces cardiac fibrosis, and promotes the expression of collagen I and III in fibroblasts, whereas reducing the cytoplasmic Ca^2+^ by inhibiting extracellular Ca^2+^ inflow attenuates Ang II-induced cardiac fibrosis^11^. Similar studies found that reducing the cytoplasm Ca^2+^ by the inhibition of T/L-type calcium channel, N-type calcium channel, or Orai1 store-operated calcium entry, also inhibits fibrosis^12–15^, raising the prospect for pharmacologic intervention of cardiac fibrosis by inhibiting Ca^2+^ signals.

Neurite outgrowth inhibitor proteins (Nogo) belong to the reticulon protein family, localizing on endoplasmic reticulum (ER) membrane with a signal peptide at the carboxy terminal. Three splicing isoforms of Nogo protein family, Nogo-A, Nogo-B, and Nogo-C, have been identified with same carboxy terminal but no homology at amino terminal^16,17^. Among three Nogo isoforms, Nogo-C is the shortest one and expresses in multiple tissues and cells, including neuron, skeletal muscle cells, vascular smooth muscle cells, liver, and heart^18,19^. Previous studies have shown that Nogo-C inhibits hepatoma carcinoma cell proliferation^20^, induces HEK293 cell apoptosis^21^, and hinders axonal re-extension after trauma^22^. Our previous study has shown that Nogo-C is upregulated during MI and the increased Nogo-C instigates cardiomyocyte apoptosis, while knockout of Nogo-C ameliorates cardiac function after MI^19^. However, it is not clear whether Nogo-C functions in cardiac fibroblast or contributes to post-MI cardiac fibrosis.

In the present study, by employing both in vivo MI models and in vitro primary cultured cardiac fibroblasts, we found that Nogo-C had a crucial role in regulating post-MI fibrosis. We showed that Nogo-C was upregulated in post-MI fibrosis models and in TGF-β1- or Ang II-stimulated cardiac fibroblasts, whereas overexpressing Nogo-C in cardiac fibroblast induced increased expression of ECM proteins. Functionally, we found that Nogo-C deficiency prevented the expression of ECM proteins in TGF-β1- or Ang II-stimulated cardiac fibroblast and in post-MI hearts, as well as ameliorated post-MI cardiac function. Mechanistically, we demonstrated that Nogo-C elevated intracellular Ca^2+^ in fibroblast by increasing Ca^2+^-leakage from ER through the stabilization of the ER Ca^2+^-leakage channel Sec61α protein. Buffering intracellular Ca^2+^ or inhibiting Sec61α blocked Nogo-C-induced pro-fibrogenic protein expression. Thus, our study reveals a novel mechanism underlying post MI fibrosis and may shed a light on clinical therapeutic strategy for cardiac remodeling related heart diseases.

## Materials and methods

### Ethics statement

This study was carried out in strict accordance with the recommendations of the Guide for the Care and Use of Laboratory Animals of the Chinese Association for Laboratory Animal Science. All procedures of animal handling were approved by the Animal Care Committee of Peking University Health Science Center. Every effort was made to minimize animal suffering.

### Mouse MI model

Nogo-C knockout mice (Nogo-C^−/−^) were generated by TALEN technique with C57BL/6 background. 8 base pairs of exon 1c, the specific exon of Nogo gene, were chopped to induce a frame-shift mutation^19^. Mouse MI model was established with Nogo-C^−/−^ and wild-type C57BL/6 male mice at the age of 8–12-week as previously described^23^. Mice were anesthetized by intraperitoneal injection of pentobarbital sodium (60 mg/kg). The fourth inter costal space over the left chest was exposed, the heart was rapidly squeezed out and the left anterior descending coronary artery (LAD) was ligated by a 6 sterile silk suture. The heart is immediately placed back into the intrathoracic space. After the incision is closed, one dose of buprenorphine (0.1 mg/kg) is administered subcutaneously as an analgesic and twice daily thereafter for 72 h. Sham subjects underwent same operation without the LAD ligation. Echocardiography was performed and hearts were collected at 28 days after the operation.

### Echocardiography

Mouse echocardiography was performed with the Vevo770 RMV-707B (Visual Sonics, Toronto, Ontario, Canada) echocardiography system. Mice were lightly anesthetized with pentobarbital sodium (25 mg/kg). 2-dimensional and M-mode images were obtained both in the long- and short-axis views. Measurement of ejection fraction (EF) and fractional shortening (FS) were performed on acquired images of 3 independent cardiac cycles from each mouse.

### Histological analyses

Heart samples were fixed with 4% paraformaldehyde and imbedded with paraffin wax. The Heart slides were stained with hematoxylin and eosin for structural evaluation and with sirius red for the evaluation of cardiac interstitial fibrosis. Collagen deposition was determined by calculating the percentage of sirius red–positive area over the total area analyzed. Images were captured on the NDP.view2 imaging workstation. Image ProPlus 6.0 was used for image analysis of the entire cardiac sections.

### Isolation and culture of rat neonatal cardiac fibroblasts

Ventricles of Sprague–Dawley rats postnatal 1–2 days were cut off and digested in HBSS solution (KCl 0.4 g/L, KH_2_PO_4_ 0.06 g/L, NaHCO_3_ 0.35 g/L, NaCl 8 g/L, Na_2_HPO_4_ 12H_2_O 0.12 g/L, glucose 1 g/L, pH 7.4) containing 0.05% type II collagenase (Worthington, USA) and 0.1% trypsin (Invitrogen, USA). Supernatant was collected and centrifuged. Cells were pre-plated for 2 h and the supernatant containing cardiomyocytes was removed. Cardiac fibroblasts were cultured with DMEM nutrient medium containing 15% fetal bovine serum (HyClone), then digested and passaged after another 24–48 h culture. The second passage was used for adenovirus transfection or for other experiments. In subsequent experiments, cells cultured to 70–80% confluence were stimulated with 10 ng/ml TGF-β1 (Sigma, USA) or 0.1 μM Ang II (Tocris, UK) for 48 h.

### Plasmid and adenovirus constructions

Constructions of adenovirus were as described previously^19^. Briefly, the amplified product of Nogo-C was inserted into pENTR/TEV/D-TOPO vector (Invitrogen), and constructed product was recombined with pAd/CMV/V5-DEST vector (Invitrogen). Sec61α-shRNA (5′-GAGAGGAAGATTCAGTTTA-3′, rat) and Nogo-C-shRNA sequence was inserted into pENTR^TM^/U6 vector and then recombined with pBLOCK-iT^TM^-DEST vector. Adenovirus was produced with Adenoviral Expression System (Invitrogen) and purified using Vivapure Adeno-PACK Kit (Sartorius, Göttingen, Germany).

### Western blot

Proteins were extracted from cardiac fibroblasts or mouse myocardium by RIPA lysis buffer (Applygen, China) containing protease inhibitor cocktail (Sigma, Santa Clara, CA, USA). Total protein concentrations were measured by BCA assay (Pierce, USA). Proteins were then separated by SDS-PAGE and transferred to PVDF membranes (Merck Millipore, Germany). Membranes were incubated with primary antibodies collagen type I, Fibronectin (FN), Sec61α (Abcam, Cambridge, MA), TGF-β1, a-smooth muscle actin (α-SMA), connective tissue growth factor (CTGF) (Santa Cruz Biotechnology, CA, USA), α-Tubulin (Bioworld, USA), or Nogo-C (Abmart, China) and then incubated with secondary antibodies goat anti-rabbit IgG, goat anti-mouse IgG, rabbit anti-goat IgG (Biodragon, China), or mouse anti-rabbit IgG LCS (Abbkine, California, USA). Immunoblots were evaluated using the Chemi Doc XRS + instrument (Bio-RAD, Hercules, CA, USA).

### Co-immunoprecipitation (Co-IP)

Cardiac fibroblasts were harvested and lysed in RIPA lysis buffer containing protease inhibitor cocktail and 1 mM PMFS. Cell lysates were centrifuged and proteins in the supernatant were collected. Proteins were pre-incubated with Protein A/G Agarose Beads (CMCTAG, Milwaukee, USA) for 3 h and then centrifuged, the supernatant were collected and incubated with Sec61α, Nogo-C or rabbit IgG (Biodragon, China) antibodies at 4 °C overnight. Subsequently, the products were incubated with Protein A/G Agarose Beads at 4 °C for 3 h. The beads were washed three times with RIPA lysis buffer, then three times with wash buffer (Tris 0.1211 g/100 ml PH8.0, NaCl 0.8775 g/100 ml, Tritonx-100 1 ml/100 ml, EDTA 0.03722 g/100 ml) and the precipitated proteins were mixed with loading buffer for further analyzed.

### Ubiquitination assay

Cardiac fibroblasts were transfected with Ad-LacZ or Ad-Nogo-C adenovirus for 48 h. MG132 (10 μM) was added 12 h before the cells were harvested. Whole cell lysates were immunoprecipitated with anti-Sec61α antibody and then immunoblotted with anti-ubiquitin (Ub) antibody to evaluate the ubiquitination level of Sec61α protein.

### Immunofluorescence

Cardiac fibroblasts plated on Laser confocal petri dish were rinsed with pre-warmed PBS (0.01 M, PH7.4), fixed with 4% paraformaldehyde for 10 min, and permeabilized with 0.5% Triton-100 for 15 min. Cells were then blocked with 5% goat serum for 30 min, incubated with indicated primary antibodies (1:200) at 4 °C overnight, and incubated with Alexa-fluor 488 goat-anti-rabbit IgG, or Alexa-fluor 488 goat-anti-mouse IgG 1:200 for 1 h at room temperature. PDI was used to visualize the endoplasmic reticulum, and 4, 6-diamidino-2-phenylindole 303 (DAPI, Sigma-Aldrich) to nuclei. Images were captured on a laser scanning confocal microscopy (TCS SP5 II, Leica).

### Measurement of cytosolic Ca^2+^ concentration

Cardiac fibroblasts planted on the petri dish were incubated with 2 μmol L^−1^ Fura-2/AM (a fluorescent Ca^2+^ probe) in 1.8 mM Ca^2+^ Tyrode buffer (CaCl_2_ 1.8 mM, NaCl 137 mM, HEPES 20 mM, KCl 5.4 mM, MgCl_2_·6H_2_O 1.2 mM, NaH_2_PO_4_·2H_2_O 1.2 mM, D-Glucose 10 mM, pH 7.4) for 30 min at 37 °C. Cells were then washed twice with Tyrode buffer at room temperature. The petri dish with cardiac fibroblasts were mounted onto the stage of the microscope and fluorescence densities of the cells were recorded at room temperature. Fluorescence images were taken by using an inverted fluorescence microscope IX71 (Olympus) with excitation at 340 and 380 nm and emission at 515 nm. Each view repeated 3 times (800 ms for each cycle) and average data of the three images were taken. Cytosolic Ca^2+^ concentration was calculated in individual cells by 340/380 nm fluorescence ratio. Data from 30 individual cells were collected per experiment.

For ER calcium release experiment, cardiac fibroblasts planted on the petri dish were loaded with 2.5 μmol L^−1^ Fluo-4 AM in 1.8 mM Ca^2+^ Tyrode buffer at 37 °C for 20 min, then washed twice with Tyrode’s solution. Cardiac fibroblasts in Ca^2+^ free Tyrode buffer were mounted onto the stage of the microscope and the Fluo-4 AM fluorescent signals were monitored at room temperature. Images were taken by Zeiss LSM 710 confocal microscope with excitation at 488 nm and emission at 505–530 nm. 300 frames of 128 × 128 pixels were collected 0.244 s/frame in a bidirectional scanning mode. Caffeine (10 mmol/L) was then added rapidly. ER Ca^2+^ content was assessed by measuring the peak amplitude of the cytosolic Ca^2+^ transient which was indexed by the normalized fluorescence, F/F_0_ (F_0_ refers to the level before caffeine was added). The time course of Ca^2+^ release was recorded and the Ca^2+^ reuptake kinetics (Tau) of the exponential part of the decay phase was assessed.

### Statistical analyses

All results were presented as the mean ± SEM. Statistical significance of differences between groups was analyzed by unpaired *t*-test or one-way ANOVA followed by SNK (Student–Newman–Keuls) when multiple groups were compared. *P* < 0.05 was considered statistically significant. GraphPad Prism software was used for all statistical analyses.

## Results

### Nogo-C is upregulated in post-MI mouse hearts and Ang II- or TGF-β1-stimulated rat cardiac fibroblasts

**Fig. 1:**
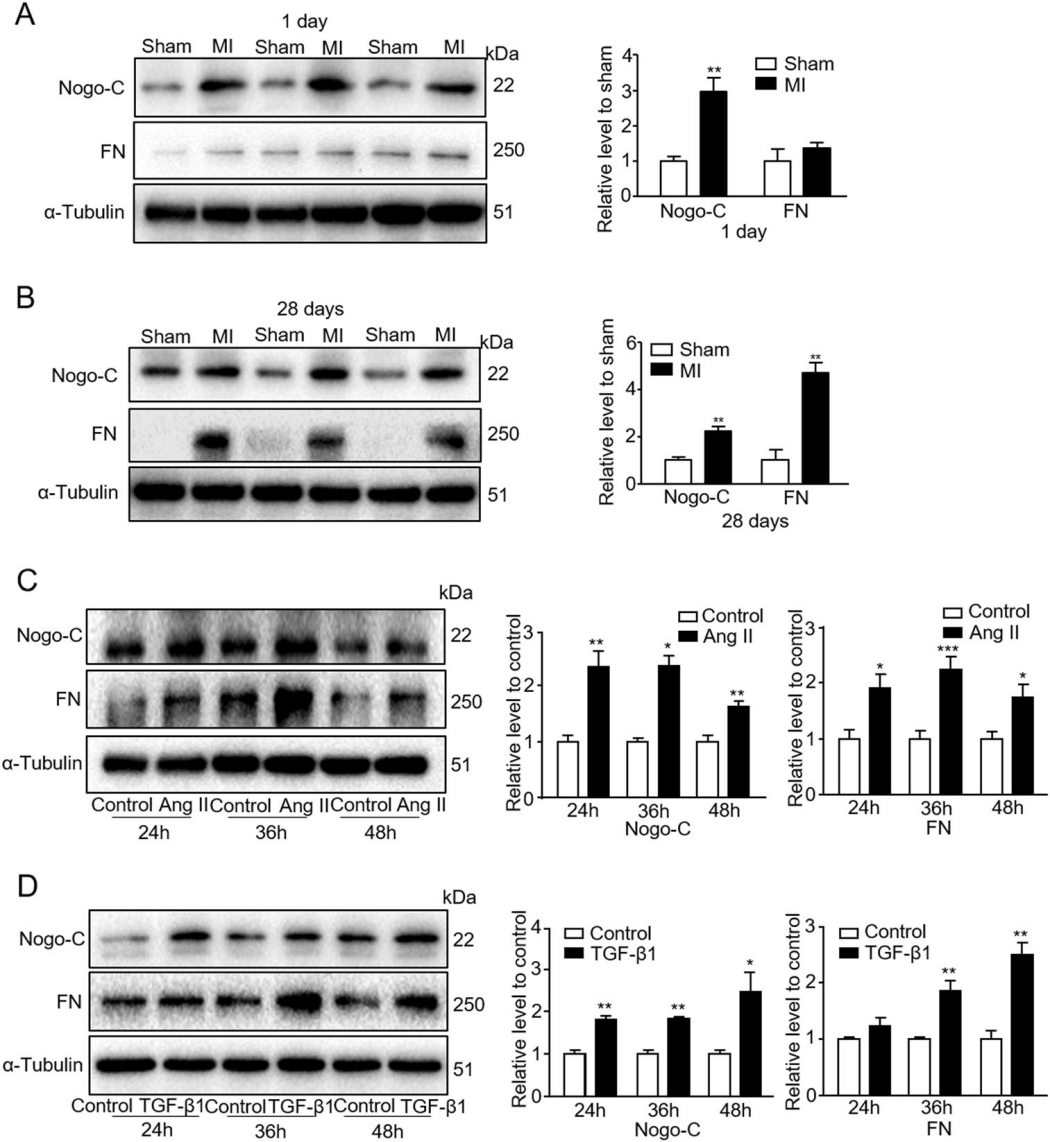
Nogo-C is upregulated in post MI mouse hearts and rat cardiac fibroblasts treated with Ang II or TGF-β1. **a**, **b** Western blot showing Nogo-C and fibronectin (FN) protein levels in sham and myocardial infarction (MI) mouse hearts 1day **(a)** or 28 days **(b)** after operation. *n* = 3 mice in every group. **P < 0.01 versus sham mice. **c** Western blot and average data showing Nogo-C and FN protein levels in rat neonatal cardiac fibroblasts treated with 0.1 μM Ang II for indicated times. **d** Western blot and average data showing Nogo-C and FN protein levels in rat neonatal cardiac fibroblasts treated with 10 ng/ml TGF-β1 for indicated times. *n* = 3 independent experiments. *P < 0.05, **P < 0.01, ***P < 0.001 versus untreated control cells

We previously showed that in post-MI mouse hearts, Nogo-C protein increased and the up-regulated Nogo-C mediates cardiomyocyte apoptosis, subsequently causing cardiac dysfunction during MI^19^. Cardiac fibroblasts are majority cells in post-MI hearts and contribute largely to post-MI cardiac dysfunction and heart failure. We hypothesize that Nogo-C is involved in the regulation of cardiac fibrosis. To test this, we measured Nogo-C protein level in post-MI mouse hearts. Nogo-C protein increased in mouse hearts 1 day after MI compared with sham control mouse hearts (Fig. 1a), in consistency with our previous observation. Importantly, the high level of Nogo-C protein lasted at least to 28 days after MI, along with the overtly increased expression of profibrogenic protein FN (Fig. 1b), the important component of ECM proteins which is essential for cell adhesion and growth^24,25^, indicating the time when the heart appears obvious fibrosis after MI.

Primary rat neonatal cardiac fibroblasts were stimulated with Ang II or TGF-β1 to establish fibrotic fibroblast models. Similar as in post-MI hearts, in cardiac fibroblasts the Nogo-C protein level was significantly increased at 24, 36, and 48 h after Ang II (0.1 μM) stimulation, compared with control cells without Ang II stimulation (Fig. 1c). FN was also increased accordingly (Fig. 1c), indicating that Ang II stimulation does induce fibrotic response in cardiac fibroblasts. Likewise, both Nogo-C and FN were up-regulated in cardiac fibroblasts stimulated with TGF-β1 (10 ng/ml) (Fig. 1d). Together, our data show that Nogo-C protein level increased both in fibrotic post-MI mouse hearts and fibrotic factor-induced cardiac fibroblasts, suggesting that Nogo-C may be involved in the pathogenesis of cardiac fibrosis.

### Nogo-C induces production of profibrogenic proteins in cardiac fibroblasts

**Fig. 2:**
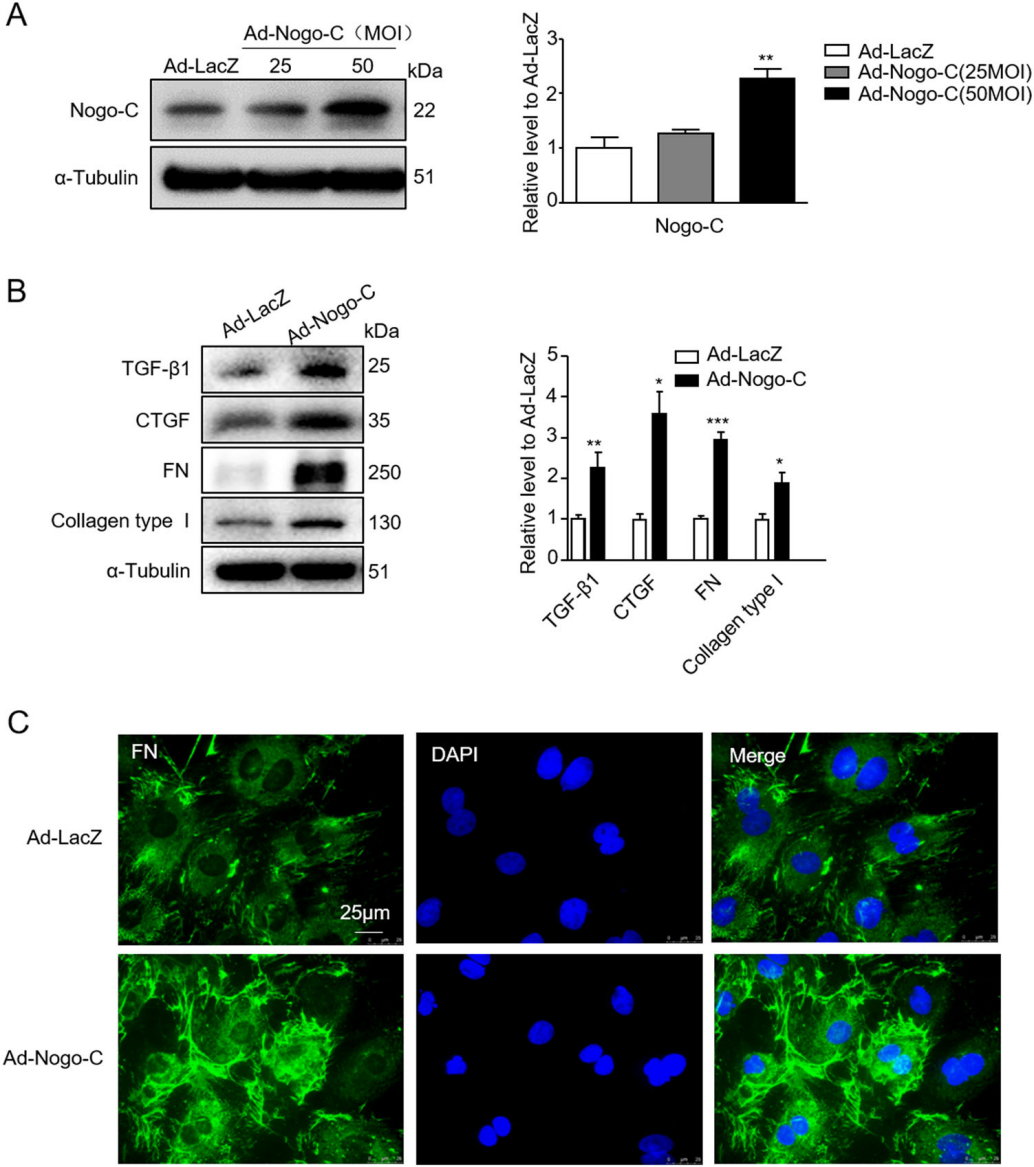
Nogo-C increases expression of profibrogenic proteins in cardiac fibroblast. **a** Western blot and average date showing the Nogo-C protein level in rat cardiac fibroblasts transfected with Ad-Nogo-C at 25 and 50 m.o.i or Ad-LacZ for 48 h. *n* = 3 independent experiments. **b** Western blot and average data of protein levels of profibrogenic proteins in cardiac fibroblasts transfected with Ad-Nogo-C or Ad-Lacz for 48 h. *n* = 3 independent experiments. *P < 0.05, **P < 0.01, ***P < 0.001 versus Ad-LacZ transfected cells. **c** Immunofluorescence staining showing FN in cardiac fibroblasts transfected with Ad-Nogo-C or Ad-LacZ for 48 h. Scale bar = 25 μm

To answer if Nogo-C is involved in the pathogenesis of cardiac fibrosis, we overexpressed Nogo-C in rat cardiac fibroblasts by adenovirus-mediated Nogo-C cDNA (Ad-Nogo-C) transfection. Ad-Nogo-C at 50 m.o.i significantly increased cellular Nogo-C protein to 2.3-fold of control cells (Fig. 2a), parallel of the increased Nogo-C protein level in post-MI hearts and in fibrotic factor-induced fibroblasts. We then measured the effect of Nogo-C on the expression of TGF-β1 and connective tissue growth factor (CTGF), crucial fibrosis-promoting cytokines regulating ECM synthesis during interstitial fibrosis. Overexpression of Nogo-C increased the protein levels of both TGF-β1 and CTGF, compared to control fibroblasts (Fig. 2b). Also, Nogo-C increased protein levels of FN and collagen type I, the most predominant component of ECM (Fig. 2b). Immunofluorescence staining images showed that FN on plasma membrane of Nogo-C expressing fibroblasts was significantly increased as compared with control cells (Fig. 2c).

**Fig. 3:**
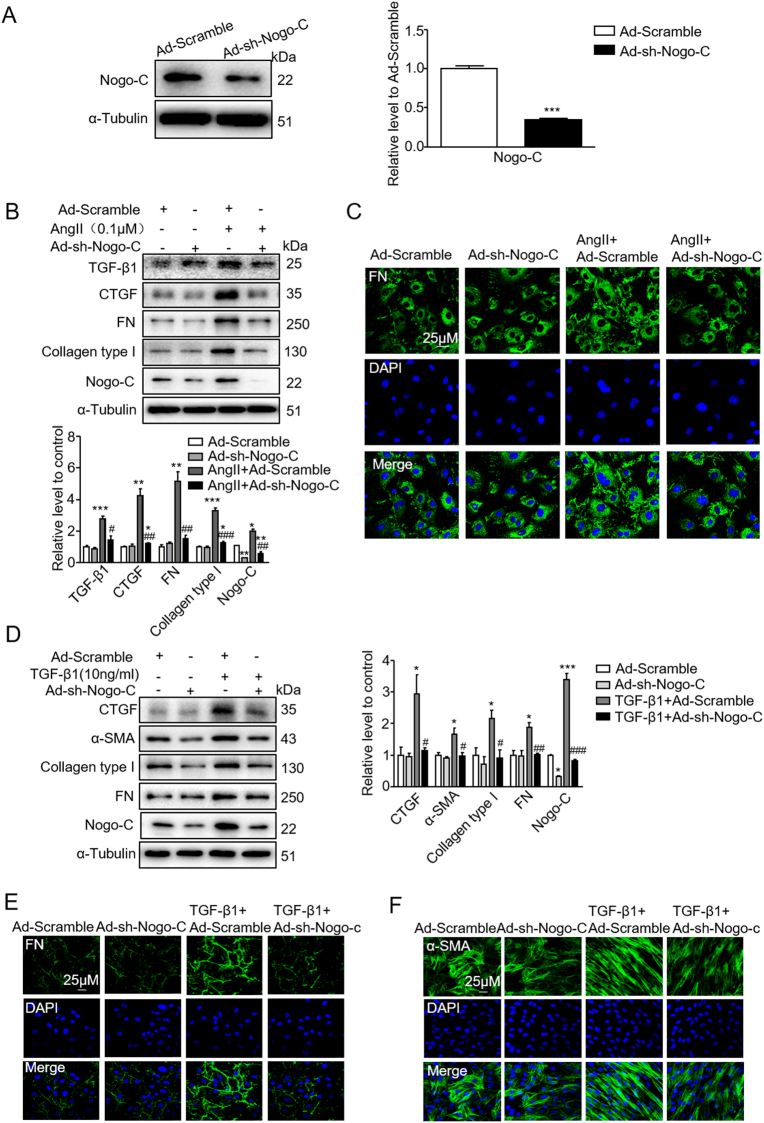
Knockdown of Nogo-C inhibits Ang II or TGF-β1-mediated expression of profibrogenic proteins in cardiac fibroblasts. **a** Nogo-C protein level in cardiac fibroblasts transfected with Ad-sh-Nogo-C or Ad-scramble for 72 h. *n* = 3 independent experiments. **b** Western blot and average date showing profibrogenic proteins CTGF, FN, collagen type I and TGF-β1 in cardiac fibroblasts transfected with Ad-sh-Nogo-C or Ad-scramble with/without Ang II (0.1 μM) stimulation. **c** Immunofluorescence staining of FN in cardiac fibroblasts transfected with Ad-sh-Nogo-C or Ad-scramble with/without Ang II stimulation. Scale bar = 25 μm. **d** Western blot and average date showing profibrogenic proteins CTGF, FN, collagen type I and α-SMA in cardiac fibroblasts transfected with Ad-sh-Nogo-C or Ad-scramble with/without TGF-β1 (10 ng/ml) stimulation. **e**, **f** Immunofluorescence staining of FN **(e)** or α-SMA **(f)** in cardiac fibroblasts transfected with Ad-sh-Nogo-C or Ad-scramble with/without TGF-β1 stimulation. *n* = 3 independent experiments. Scale bar = 25 μm. *P < 0.05, **P < 0.01, ***P < 0.001 vs Ad-scramble. ^#^*P* < 0.05, ^##^*P* < 0.01, ^###^*P* < 0.001 vs Ang II or TGF-β1 groups

We next down-regulated Nogo-C in cardiac fibroblasts by transfecting adenovirus containing Nogo-C short hairpin RNA (shRNA) (Ad-sh-Nogo-C). The protein level of Nogo-C was reduced in fibroblasts transfected with Ad-sh-Nogo-C to 30% of that in scramble control cells (Fig. 3a). While Ang II stimulation increased profibrogenic proteins TGF-β1, CTGF, FN, and collagen type I in scramble fibroblasts, knockdown of Nogo-C prevented Ang II-induced increase of profibrogenic proteins (Fig. 3b). The blockage of the elevated expression of FN on plasma membrane induced by Ang II in Nogo-C knockdown fibroblast was also visualized by immunofluorescence staining with FN antibody (Fig. 3c). Similarly, TGF-β1 stimulation caused increased CTGF, FN, and collagen type I in scramble cardiac fibroblasts, and this effect was blocked by knockdown of Nogo-C (Fig. 3d). Immunofluorescence staining showed that the increased FN on fibroblast plasma membrane by TGF-β1 stimulation was inhibited by Nogo-C knockdown (Fig. 3e). In addition, while overexpressing Nogo-C per se had no effect on α-SMA, the contractile protein indicating the transformation from fibroblasts to myofibroblasts (Figure S1a), Nogo-C knockdown depressed TGF-β1-induced α-SMA (Fig. 3f). Collectively, our results show that overexpression of Nogo-C enhances, while knockdown of Nogo-C inhibits, expression of profibrogenic proteins in cardiac fibroblasts, suggesting that Nogo-C directly regulates fibrotic response of cardiac fibroblasts.

### Nogo-C-deficiency ameliorates post-MI fibrosis and improves cardiac function

**Fig. 4:**
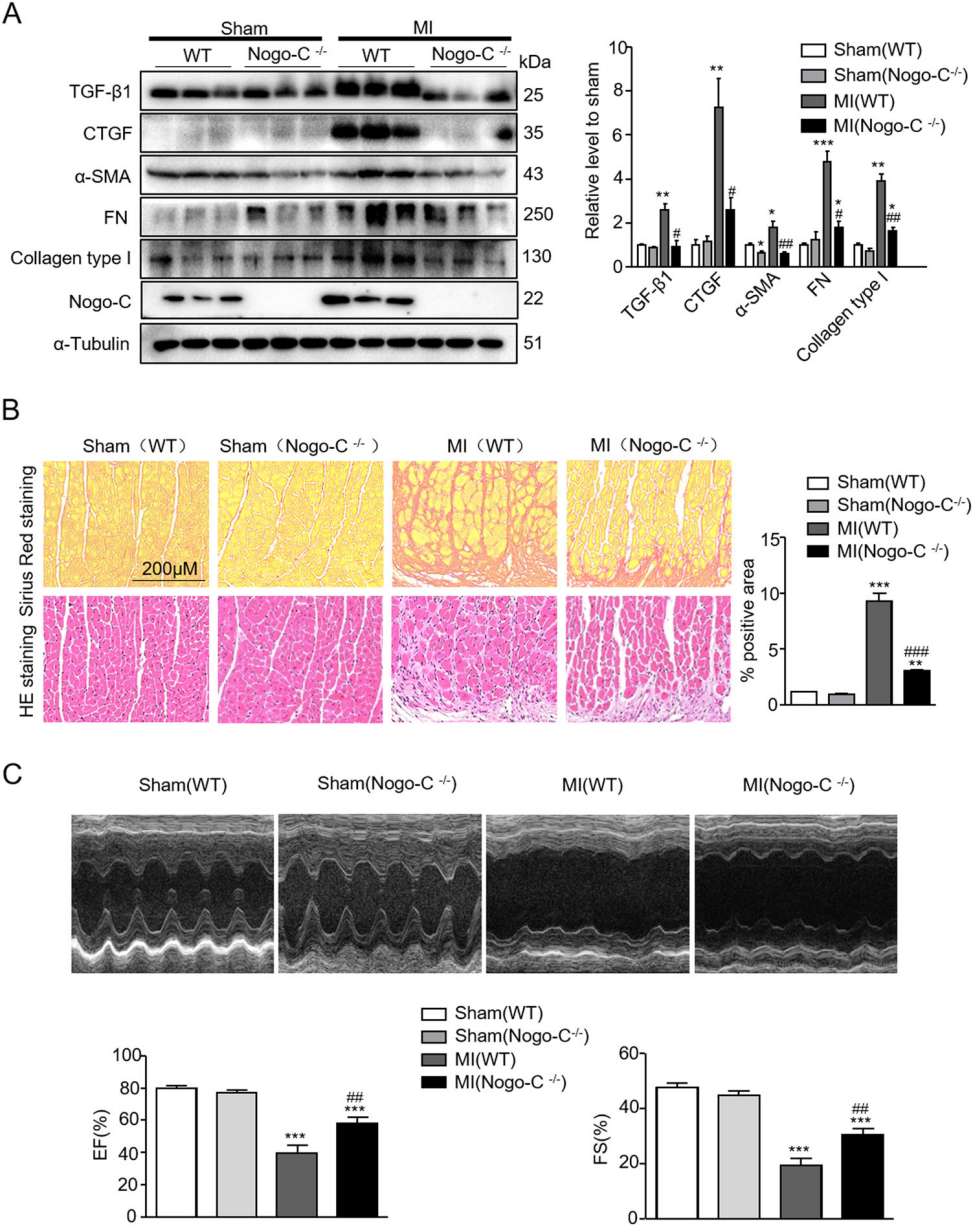
Knockout of Nogo-C inhibits post-MI fibrosis and improves cardiac function. **a** Western blot and average date showing levels of profibrogenic proteins in sham and MI hearts from wild-type and Nogo-C^−/−^ mice. *n* = 3 mice for each group. **b** Picric acid Sirius red and HE staining showing collagen deposition in MI cardiac border zone. *n* = 15 images from 3 mice for each group. Scale bar = 200 μm. **c** Representative M-mode images of echocardiography, and average data of EF (left) and FS (right) in 2-dimensional images. *n* = 8 mice for each group. *P < 0.05, **P < 0.01, ***P < 0.001 vs wild-type sham. ^#^*P* < 0.05, ^##^*P* < 0.01, ^###^*P* < 0.001 vs wild-type MI

Nogo-C is increased in fibrotic mouse hearts 28 days after MI, and increased Nogo-C protein in cardiac fibroblasts regulates expression of profibrogenic proteins. To answer if downregulation of Nogo-C in vivo functions protectively in post-MI cardiac fibrosis, we employed the Nogo-C^−/−^ mice, combined with LAD ligation-induced MI model^23^. In left ventricle of wild-type mouse hearts 28 days after MI, profibrogenic proteins α-SMA, TGF-β1, CTGF, FN, and collagen type I significantly increased, as compared with sham control hearts (Fig. 4a). In contrast, these fibrotic factors were not altered in Nogo-C^−/−^ mouse hearts at 28 days after MI, compared with Nogo-C^−/−^ sham control mice (Fig. 4a). Moreover, staining of interstitial collagen deposition by picric acid sirius red in Nogo-C^−/−^ or wild-type mouse hearts after MI showed that, Nogo-C deficiency dramatically prevented MI-caused collagen deposition as compared with wild-type littermates (Fig. 4b). Our data indicates that Nogo-C deficiency protects the heart from post-MI fibrosis.

Cardiac fibrosis largely contributes to post-MI cardiac dysfunction, we then measured cardiac function of Nogo-C^−/−^ and wild-type mice after MI. Echocardiography results showed that cardiac ejection fraction (EF) and fractional shortening (FS) in wild-type mice significantly decreased at 28 days after MI compared with sham control mice, with EF of 79.59 ± 1.574% in sham control mice versus 39.52 ± 4.65% in MI mice, and FS of 47.56 ± 1.599% in sham control mice versus 19.37 ± 2.579% in MI mice (Fig. 4c). However, Nogo-C deficiency significantly improved post-MI cardiac function to 58.1 ± 3.603% (EF) and 30.41 ± 2.273% (FS) respectively. Together, our data here further support the protective role of Nogo-C deficiency on cardiac function after MI.

### Nogo-C regulates fibrosis through Sec61 channel-mediated Ca^2+^ signals

**Fig. 5:**
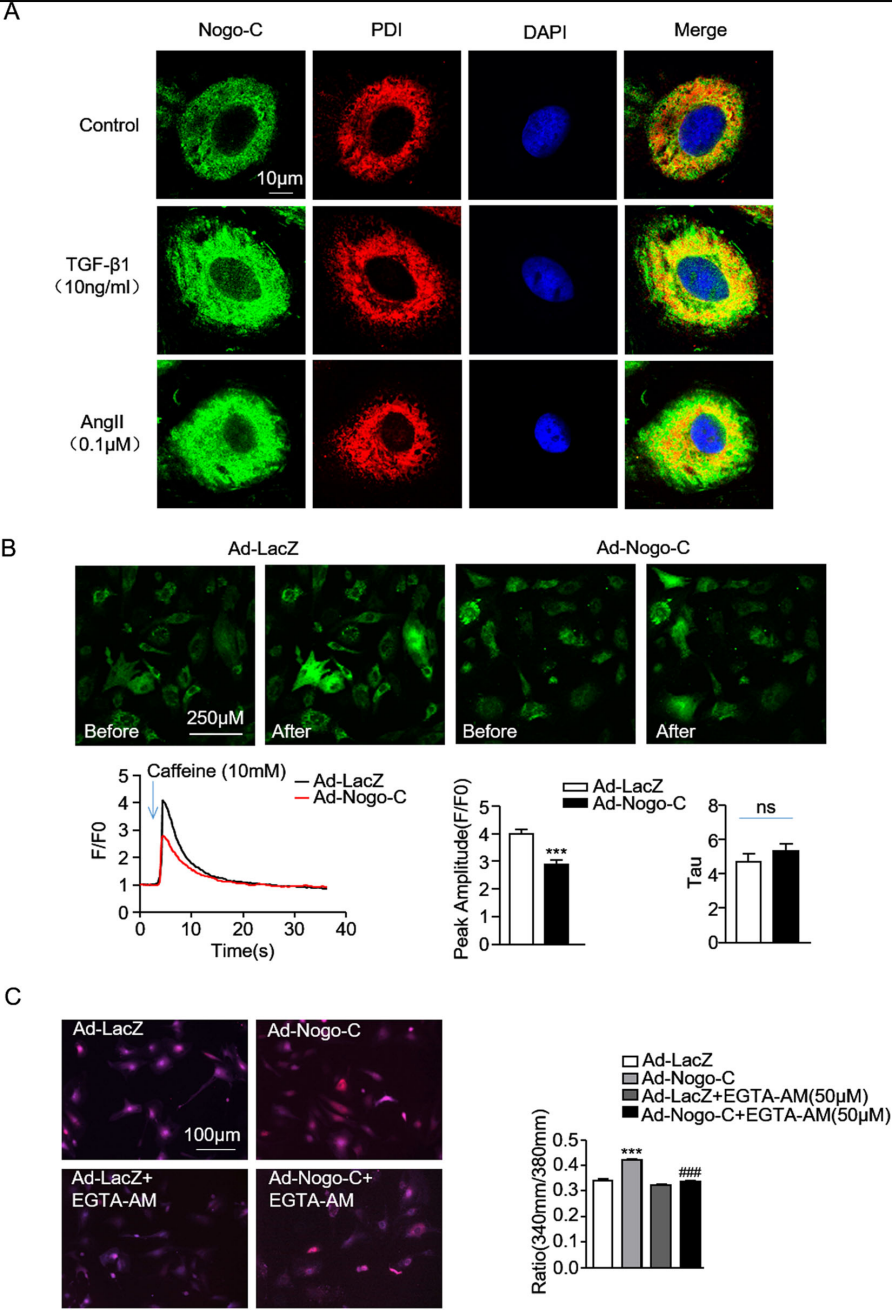

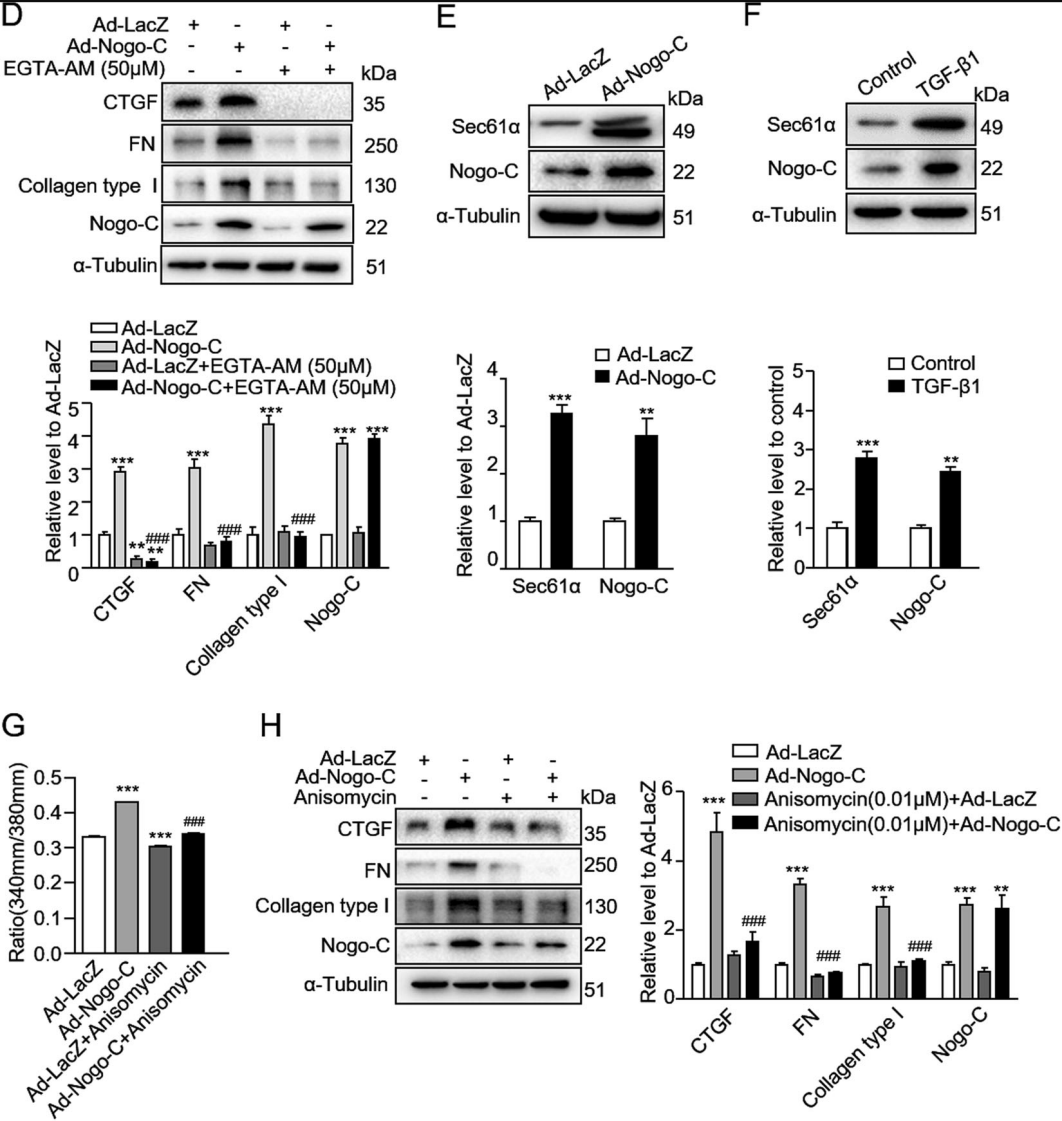
Nogo-C regulates fibrotic responses throughincreasing intracellular Ca^2+^ concentration. **a** Immunofluorescence staining showing the co-localization of Nogo-C with ER in cardiac fibroblasts with/without TGF-β1 or AngII stimulation. Scale bar = 10 μm. **b** Representative images (upper) and average data (bottom) showing the ER Ca^2+^ release in response to caffeine (10 mM) stimulation in cardiac fibroblasts transfected with Ad-Nogo-C or Ad-LacZ, indicated by Fluo-4 AM fluorescent signal. *n* *=* 12 cells from 3 independent experiments for each group. **c** Representative images (left) and average data (right) showing cytosolic Ca^2+^ concentration of cardiac fibroblasts transfected with Ad-Nogo-C or Ad-LacZ with/without EGTA-AM (50 μM) treatment. *n* *=* 30 cells from 3 independent experiments for each group. **d** Profibrogenic proteins (CTGF, FN and collagen type I) in cardiac fibroblasts transfected with Ad-Nogo-C or Ad-LacZ with/without EGTA-AM (50 μM) treatment. *n* = 3 independent experiments. **e** Sec61α protein level in cardiac fibroblasts transfected with Ad-Nogo-C. **f** Sec61α protein level in cardiac fibroblasts in the presence of TGF-β1 (10 ng/ml) for 48 h. **g** Cytosolic Ca^2+^ concentration in cardiac fibroblasts transfected with Ad-Nogo-C or Ad-LacZ with/without Sec61α inhibitor Anisomycin (0.01 μM). **h** Profibrogenic proteins in cardiac fibroblasts transfected with Ad-Nogo-C or Ad-LacZ with/without Sec61α inhibitor Anisomycin (0.01 μM). *n* = 3 independent experiments. *P < 0.05, **P < 0.01, ***P < 0.001 vs Ad-LacZ or control group. ^#^*P* < 0.05, ^##^*P* < 0.01, ^###^*P* < 0.001 vs Ad-Nogo-C group

To understand the underlying mechanism of Nogo-C on cardiac fibrosis, we at first detected the localization of Nogo-C in cardiac fibroblasts. Immunofluorescence staining showed that Nogo-C co-localized with endoplasmic reticulum (ER), as indicated by the overlapped Nogo-C fluorescent signal and ER marker PDI fluorescence in cardiac fibroblasts (Fig. 5a). Moreover, the upregulated Nogo-C in response to TGF-β1 or AngII stimulation mainly co-localized with ER (Fig. 5a). ER is the main organelle to sequestrate and release intracellular calcium, and the disturbance of calcium homeostasis plays a pivotal role in the pathogenesis of cardiac fibrosis^11^. Thus, we measured possible changes of ER calcium content in response to caffeine stimulation, as indicated by the Fluo-4 AM fluorescent density. We found that caffeine caused decreased ER Ca^2+^ release in Nogo-C overexpressing cardiac fibroblasts compared with control cells (Fig. 5b). The normalized index F/F0 (F0 refers to the level before caffeine stimulation) showed that Nogo-C overexpression decreased peak amplitude of ER Ca^2+^ release but did not change the Tau, indicating decreased ER calcium content and unaltered ER calcium uptake (Fig. 5b). We then measured the intracellular Ca^2+^ changes in response to Nogo-C. Overexpression of Nogo-C significantly increased the intracellular Ca^2+^ concentration, as indicated by the increased 340/380 nm fluorescence ratio of Fura-2/AM, the fluorescent probe of intracellular Ca^2+^, and this Nogo-C-induced intracellular Ca^2+^ elevation was blocked by EGTA-AM, the intracellular Ca^2+^ chelator (Fig. 5c). We next examined the production of profibrotic proteins CTGF, FN, and collagen type I in response to Nogo-C in the presence of EGTA-AM. Inhibition of intracellular Ca^2+^ by EGTA-AM totally blocked Nogo-C-induced increase of these profibrotic proteins without affecting Nogo-C protein level (Fig. 5d).

To understand the molecular mechanism of Nogo-C-induced increase of intracellular Ca^2+^, we checked sec61α, the ER membrane translocation protein which is also known to mediate Ca^2+^-leakage from the ER. We found that overexpression of Nogo-C in cardiac fibroblasts increased Sec61α protein level (Fig. 5e). In addition, TGF-β1 stimulation simultaneously increased Nogo-C and Sec61α protein in cardiac fibroblasts (Fig. 5f). Moreover, anisomycin, the sec61α channel inhibitor which acts on the transfer reaction of sec61α to abrogate the calcium leakage, significantly inhibited the elevated intracellular Ca^2+^ concentration (Fig. 5g) and blocked the increased CTGF, FN, and collagen type I in response to Nogo-C overexpression in cardiac fibroblasts (Fig. 5h). These results suggest that Sec61α channel-mediated intracellular Ca^2+^ signals may contribute to Nogo-C-regulated cardiac fibrosis.

**Fig. 6:**
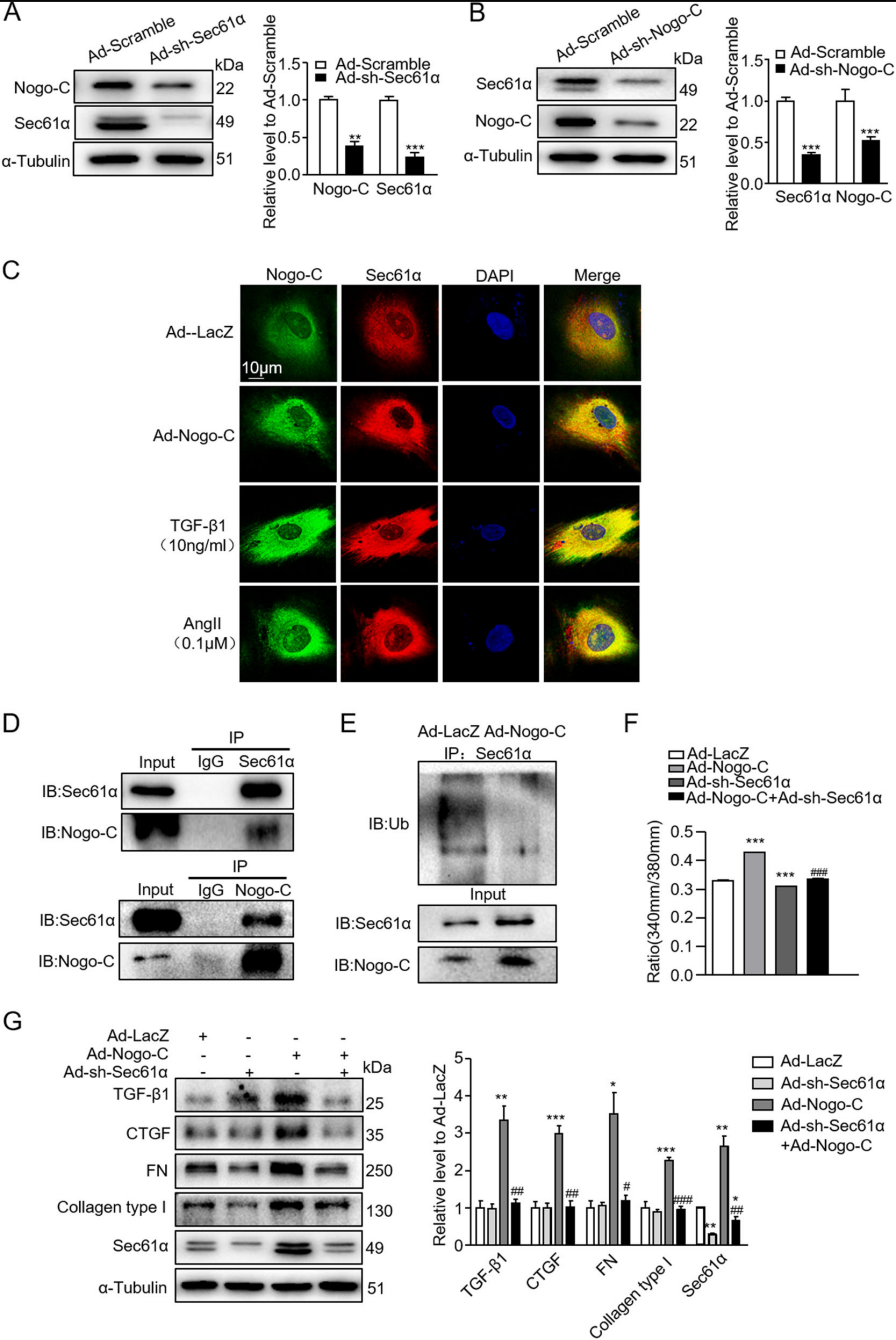

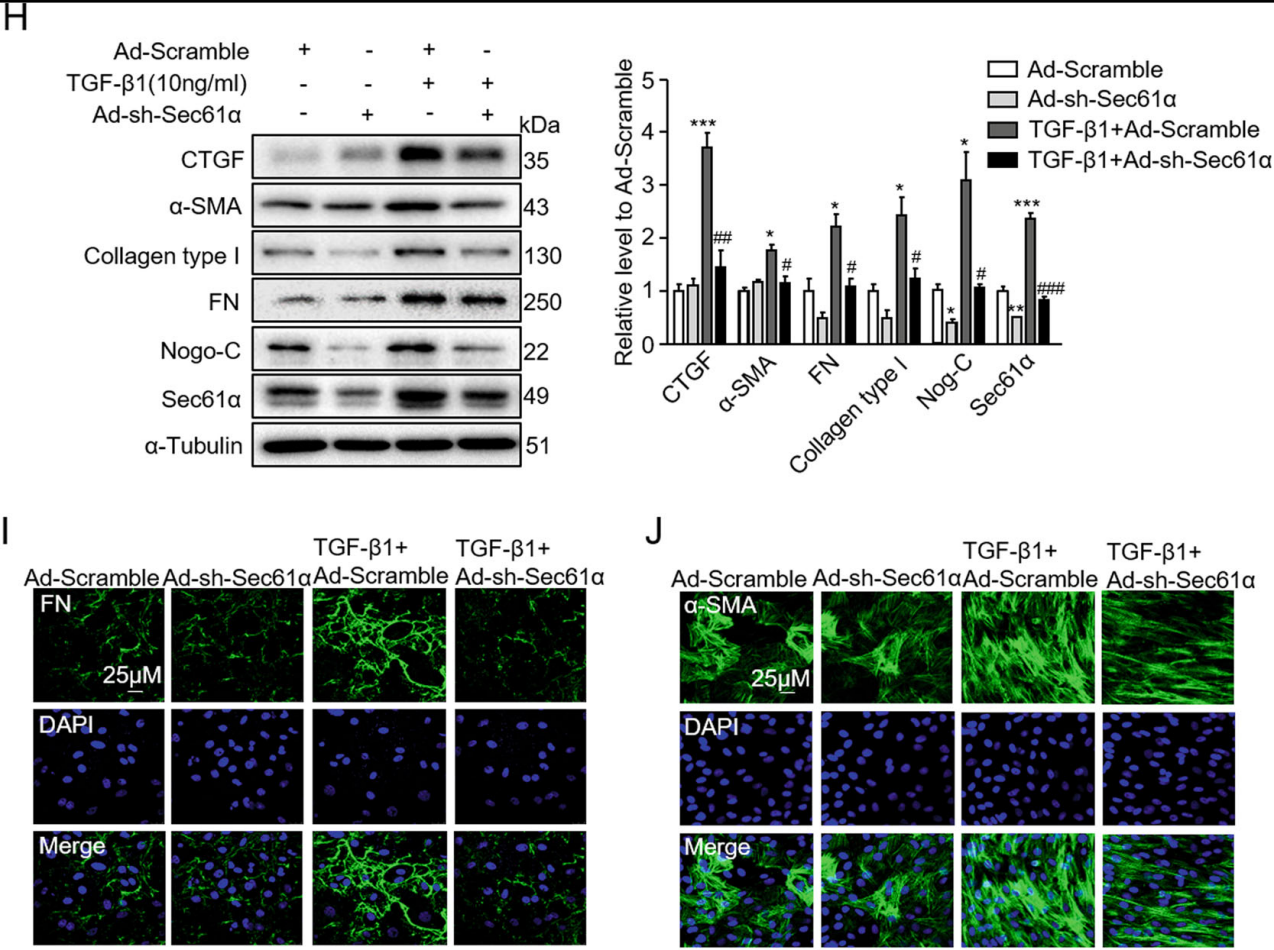
Knockdown of Sec61α inhibits Nogo-C- and TGF-β1-mediated fibrotic responses in cardiac fibroblasts. **a** Western blot and average date showing Sec61α and Nogo-C protein levels in cardiac fibroblasts transfected with Ad-sh-Sec61α or Ad-scramble for 72 h. *n* = 3 independent experiments. **b** Western blot and average date showing Nogo-C and Sec61α protein levels in cardiac fibroblasts transfected with Ad-sh-Nogo-C or Ad-scramble for 72 h. *n* = 3 independent experiments. **c** Immunofluorescence staining showing the co-localization of Nogo-C with Sec61α in cardiac fibroblasts with/without TGF-β1 or AngII stimulation. Scale bar = 10 μm. **d** CoIP of Nogo-C protein with Sec61α protein in cardiac fibroblasts. **e** Ubiquitination level of Sec61α protein in cardiac fibroblasts transfected with Ad-Nogo-C or Ad-LacZ in the presence of MG132 (10 μM). **f** Cytosolic Ca^2+^ concentration in cardiac fibroblasts transfected with Ad-Nogo-C with/without co-transfection of Ad-sh-Sec61α. **g** Western blot and average date showing levels of profibrogenic proteins TGF-β1, CTGF, FN, and collagen type I in cardiac fibroblasts transfected with Ad-Nogo-C with/without co-transfection of Ad-sh-Sec61α. **h** Profibrogenic proteins and Nogo-C in cardiac fibroblasts treated with TGF-β1 (10 ng/ml) with/without knockdown of Sec61α by Ad-sh-Sec61α transfection. *n* = 3 independent experiments. **i**, **j** Immunofluorescence staining of FN **(i)** or α-SMA **(j)** in cardiac fibroblasts in response to TGF-β1 (10 ng/ml) stimulation in the presence/absence of Ad-sh-Sec61α infection. Scale bar = 25 μm. *P < 0.05, **P < 0.01, ***P < 0.001 vs Ad-LacZ or control group. ^#^*P* < 0.05, ^##^*P* < 0.01, ^###^*P* < 0.001 vs Ad-Nogo-C or TGF-β1 group

To further pinpoint the role of Sec61α in Nogo-C-mediated cardiac fibrosis, we generated adenovirus containing Sec61α shRNA (Ad-sh-Sec61α) to knockdown Sec61α protein in cardiac fibroblasts. Interestingly, while knockdown of Sec61α declined Sec61α protein, it also decreased Nogo-C protein level (Fig. 6a). And similarly, knockdown of Nogo-C decreased both Nogo-C and Sec61α proteins in cardiac fibroblasts (Fig. 6b), indicating a possible crosstalk between the two proteins. Indeed, immunofluorescence staining showed that Nogo-C co-localized with Sec61α on endoplasmic reticulum in cardiac fibroblasts (Fig. 6c). Moreover, we found that Nogo-C interacted with Sec61α in cardiac fibroblasts (Fig. 6d), and overexpression of Nogo-C in fibroblasts reduced the amount of poly-ubiquitinated Sec61α protein (Fig. 6e), suggesting that Nogo-C interacted with Sec61α to enhance its stability by preventing Sec61α ubiquitination.

**Fig. 7:**
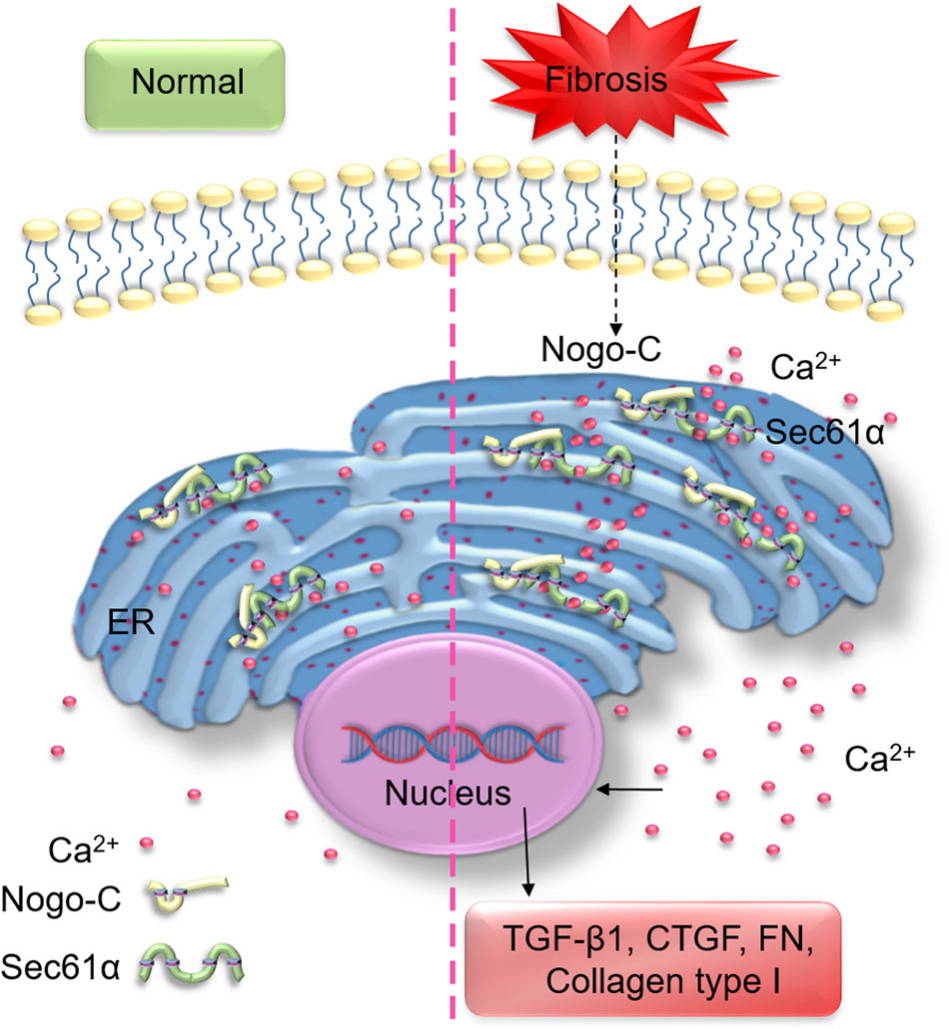
Schematic graph of Nogo-C in the regulation of post-MI fibrosis

Similar with anisomycin, Sec61α knockdown largely inhibited the elevated intracellular Ca^2+^ concentration by Nogo-C overexpression (Fig. 6f) and absolutely abolished Nogo-C-induced increase of profibrotic proteins TGF-β1, CTGF, FN, and collagen type I in cardiac fibroblasts (Fig. 6g). Consistently, Sec61α knockdown also blocked TGF-β1-induced fibrosis factors CTGF, FN, collagen type I, α-SMA, and even TGF-β1-induced increase of Nogo-C (Fig. 6h-j). Together, our results suggest that Nogo-C regulates cardiac fibrosis by increasing cytosolic Ca^2+^ concentration, at least partially through Sec61α-mediated Ca^2+^-leakage from ER (Fig. 7).

## Discussion

In the present study, we provide evidence showing that Nogo-C is a critical player of and actively involved in the pathogenesis of cardiac remodeling after MI. We found that Nogo-C was elevated in post-MI cardiac fibrotic tissue, whereas Nogo-C deficiency ameliorated cardiac fibrosis and improved cardiac function. Mechanistically, we demonstrated that the profibrotic function of Nogo-C was through the regulation of ER Ca^2+^ channel protein Sec61-mediated Ca^2+^ leakage from ER in cardiac fibroblasts.

Cardiac fibroblasts are abundant in normal hearts and constitute the majority of cardiac cells in the post-MI remodeling hearts^26,27^. After MI, cardiac fibroblasts play multifaceted roles including the activation of inflammatory reaction, paracrine signaling, and scar formation. In the injured myocardium, inflammatory factors such as free radicals and nuclear factor (NF)-κB stimulate fibroblasts to release pro-inflammatory cytokines and secrete various paracrine factors^28–31^, then the paracrine mediators further induce fibroblasts to proliferate, differentiate to myofibroblasts, and secrete ECM proteins^1,25,32,33^. The present study found that Nogo-C protein was upregulated in post-MI fibrotic hearts. We speculate that Nogo-C might be increased in post-MI fibroblasts, the prevalent cell type in post-MI hearts, and be regulated by fibrotic cytokines. Indeed, fibrotic factors TGF-β1 and Ang II upregulate Nogo-C protein in cardiac fibroblasts, supporting that Nogo-C in cardiac fibroblasts is regulated by the increased local inflammatory cytokines or fibrotic factors. Interestingly, Nogo-C per se causes increased TGF-β1 level from fibroblasts, in general agreement with the paracrine function of cardiac fibroblasts and further indicating the involvement of Nogo-C in the fibrotic response of fibroblasts.

The present study reveals that Nogo-C is actively involved in the regulation of post-MI cardiac fibrosis, Nogo-C deficiency largely inhibits the development of post-MI fibrosis and improves the post-MI cardiac function. In addition to Nogo-C, another two isoforms of Nogo family, Nogo-A and Nogo-B, have also been reported to play roles in cardiac fibrosis^34,35^. During liver fibrosis, Nogo-B inhibits hepatic stellate cell apoptosis and facilitates TGF-β-Smad2 pathway to mediate hepatic fibrosis^36,37^. However, the present study found that Nogo-C mediated the fibrotic response of cardiac fibrosis without affecting fibroblast apoptosis or proliferation (figure S1b and S1c), suggesting a distinct way of Nogo-C from Nogo-B in the regulation of cardiac fibrosis. Nogo family members are ER proteins, and the current study also confirmed that Nogo-C localized on ER in cardiac fibroblasts. As the main intracellular calcium store, ER is critical for the maintenance of calcium homeostasis^38–40^. We found that overexpression of Nogo-C in cardiac fibroblasts increased intracellular calcium. Actually, intracellular Ca^2+^ is an important signal in TGF-β-mediated fibrosis process^11,41–43^. Inhibition of intracellular calcium by EGTA-AM totally blocked Nogo-C-induced increase of fibrotic factors without affecting Nogo-C protein level, supporting that increased intracellular calcium mediates Nogo-C-induced fibrotic responses.

Sec61α is also an ER membrane protein and has been primarily found to mediate translocation of polypeptide chains into the cisterns of ER during protein biogenesis. Lately, Sec61α has been reported to conduct Ca^2+^-leakage from the ER^44–46^. The current study found that the upregulation of Sec61α accompanied with the increased Nogo-C protein in both Nogo-C overexpressing and in TGF-β1-stimulated cardiac fibroblasts, whereas inhibition of Sec61α by either Sec61 channel inhibitor anisomycin or knockdown of Sec61α inhibited Nogo-C elevated cytosolic Ca^2+^ concentration and blocked Nogo-C- and TGF-β1 induced fibrotic responses. We further demonstrated that Nogo-C interacted with and stabilized Sec61α protein through inhibiting its ubiquitination, suggesting that Nogo-C increases cytosolic Ca^2+^ concentration through increased ER Ca^2+^ leakage due to the stabilized Sec61α protein. Intriguingly, we found that while knockdown of Nogo-C caused the decrease of Sec61α, knockdown of Sec61α also led to a decrease of Nogo-C, suggesting that these two ER membrane proteins possibly stabilize each other. However, the exact mechanism underlying the mutual regulation of Nogo-C and Sec61α merits further investigation.

We found that overexpressing Nogo-C in cardiac fibroblasts caused increased fibrotic cytokines (TGF-β1 and CTGF) and ECM deposition (FN and collagen type I), and knockdown of Nogo-C alleviated fibrotic factors-induced fibrotic responses. α-SMA in combination with fibrotic factors are commonly used for the identification of fibrotic responses in cardiac fibroblasts. However, we found that overexpressing Nogo-C per se had no effect on α-SMA expression while knockdown of Nogo-C prevented TGF-β1-induced α-SMA upregulation. Although the mechanism is not yet clear, a possibility is the divergent downstream pathways in Nogo-C- and TGFβ1- mediated fibrotic responses. Considering that the activated myofibroblasts exhibit extensive endoplasmic reticulum, allowing it to synthesize and secrete ECM proteins for the repair of injured myocardium^47,48^, it is reasonable to speculate that the increased Nogo-C, the endoplasmic reticulum protein, also relates to this process by its mutual regulation with Sec61. In fact, the Sec61 channel is also associated with fibrotic protein translocation and synthesis in addition to its effect on Ca^2+^ efflux from the ER^49^. mRNAs of two collagen type I subtypes are delivered to the protein translocation channel through the 5′ UTRs structure 5′SL/ LARP6 (La-domain ribonucleoprotein 6) complex, and the association of Sec61 translocon with the 5′SL/LARP6 complex is required for this translocation and for the collagen synthesis^49^. Thus, Nogo-C-mediated Sec61 stabilization may contribute to the pathogenesis of post-MI cardiac fibrosis by increasing Ca^2+^ leakage from the ER as well as increasing the synthesis of fibrotic factors. Nevertheless, whether Nogo-C-induced cardiac fibrosis is also through Sec61-mediated polypeptide translocation and synthesis needs further intensive study.

In conclusion, the current study identifies a novel role of Nogo-C in the pathogenesis of post-MI cardiac fibrosis (Fig. 7g). Nogo-C is upregulated during post-MI cardiac fibrosis; the upregulated Nogo-C interacts with ER Ca^2+^-leakage channel Sec61α to stabilize Sec61α protein, thus causing increased Ca^2+^-leakage from ER and subsequently increased cytosolic Ca^2+^ concentration. Inhibition of cytosolic Ca^2+^ or inhibition of Sec61α channel in fibroblasts blocks Nogo-C and fibrotic cytokines-induced fibrotic responses; more importantly, Nogo-C deficiency ameliorates post-MI cardiac fibrosis and improves cardiac function. Thus, our findings of the mechanism underlying Nogo-C- mediated cardiac fibrosis provide potential therapeutic targets for post-MI fibrotic remodeling related cardiac diseases.

## Electronic supplementary material

Figure S1

Supplemental information
