# Supplementary figures and images for: Nogo-C regulates post myocardial infarction fibrosis through the interaction with ER Ca2+ leakage channel Sec61α in mouse hearts
Source: Cell Death Dis. 2018 May 23;9(6):612. doi: 10.1038/s41419-018-0598-6 (PMC5966439; doi:10.1038/s41419-018-0598-6)

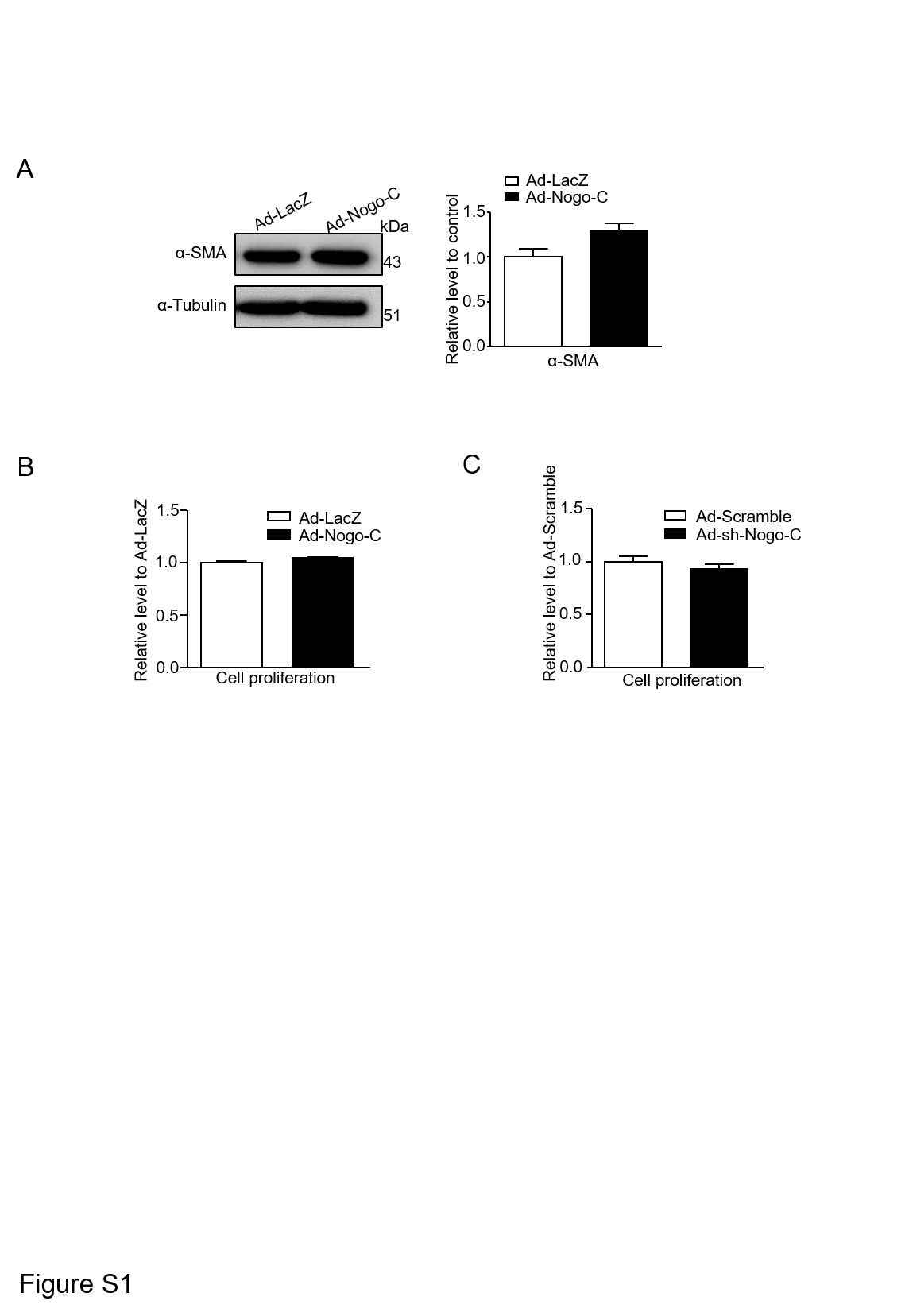

Supplement: Supplementary file 1 — Figure S1 [file 41419_2018_598_MOESM1_ESM.tif]
