# Supplementary material for: Nogo-C regulates post myocardial infarction fibrosis through the interaction with ER Ca2+ leakage channel Sec61α in mouse hearts
Source: Cell Death Dis. 2018 May 23;9(6):612. doi: 10.1038/s41419-018-0598-6 (PMC5966439; doi:10.1038/s41419-018-0598-6)
Supplement: Supplementary file 2 — Supplemental information [file 41419_2018_598_MOESM2_ESM.docx]

**Supplementary Information**

**Supplementary materials and methods**

**CCK-8 cell proliferation assay**

Cell proliferation was determined by using a cell counting kit-8 (CCK-8) (Dojindo, Kumamoto, Japan) assay. Approximately 5000 cardiac fibroblasts were plated on each well of a 96-well plate in 100 μl of DMEM medium. Cells were cultured to 60%-70% confluence and transfected with Ad-Nogo-C or Ad-LacZ for 48h, or with Ad-sh-Nogo-C or Ad-scramble for 72h. 10μl of CCK-8 was added to each well and the cell proliferation was measured 1h later at 450 nm using a microplate reader (Bio-Rad, USA).

**Supplementary figure legend**

**Figure S1. (a)** Western blot and average date showing α–SMA protein levels in cardiac fibroblasts transfected with Ad-Nogo-C or Ad-LacZ for 48 hours. *n*=3 independent experiments. **(b) (c)** Cell proliferation by CCK-8 assay in cardiac fibroblasts transfected with Ad-Nogo-C or Ad-LacZ **(b)** and in cardiac fibroblasts transfected Ad-sh-Nogo-C or Ad-Scramble **(c)**. *n*=3 independent experiments.
